# Work-related musculoskeletal disorders in vulnerable populations: what are the most common body parts affected?

**DOI:** 10.1186/s12889-023-16570-2

**Published:** 2023-08-25

**Authors:** Marcele Pereira Silvestre Gotardelo, Allana Lima Moreira Rodrigues, Fernando Rodrigues Peixoto Quaresma, André Pontes-Silva, Erika da Silva Maciel

**Affiliations:** 1 Postgraduate Program in Teaching in Science and Health, Universidade Federal do Tocantins (UFT), Palmas, TO Brazil; 2 Postgraduate Program in Physical Therapy (PPGFT), Department of Physical Therapy (DFisio), Federal University of São Carlos (UFSCar), Washington Luis Road, Km 235, São Carlos, SP 13565-905 Brazil

**Keywords:** Cumulative trauma disorders, Public health, Quality of life, Musculoskeletal disorders

## Abstract

**Background:**

Studies investigating vulnerable populations have shown that work-related musculoskeletal disorders have a negative impact on quality of life. However, no study has examined the body regions commonly affected by work-related musculoskeletal disorders in vulnerable populations.

**Objective:**

To describe the body regions commonly affected by work-related musculoskeletal disorders in vulnerable populations.

**Methods:**

Cross-sectional study. We used the ABEP questionnaire, the World Health Organization Quality of Life, the Nordic Musculoskeletal Disorders Questionnaire, the Perceived Stress Scale, and a self-report questionnaire to determine morning-evening in human circadian rhythms (chronotype assessment). To reduce the possibility of information bias, we provided prior training in the use of the instruments and created an electronic database that was filled out in duplicate (in cases of disagreement, a third researcher was consulted). We tested the normality of the data using the Shapiro–Wilk test.

**Results:**

The sample consisted of 132 participants, but there was a sample loss of 41.6% (final sample *n* = 77). We observed the predominance of those who worked from 6 to 8 h/day, rest of 1 h during the working day, from 1 to 10 years of service and only 1 employment relationship. Regarding the quality of life, we observed a worse result in the domain related to the environment, as well as a stress level of 15.43 (± 7.52) with a maximum of 30. Finally, we observed the presence of pain self-reported by the artisanal fishermen in several regions of the body, lumbar being the most mentioned.

**Conclusion:**

The neck, shoulders, arms, elbows, forearms, wrists, back, lumbar spine, and lower limbs are the most common parts of the body affected by work-related musculoskeletal disorders in artisanal fishermen.

## Introduction

The relationship between man and work is a dualism of contradictions: happiness in his professional activity and frustration for the same reason. When working conditions are unfavorable, the organism is affected [1, 2]. It is estimated that 2.3 million workers die each year as a result of accidents and work-related diseases [3]. Work-related musculoskeletal disorders are one of the most common chronic non-communicable diseases in Latin America. These problems occur primarily in low- and middle-income countries (e.g., Brazil), where the lack of a right to health and effective policies undermine the quality of life of vulnerable populations (e.g., artisanal fishermen) [4].

Work-related musculoskeletal disorders have been identified as notifiable diseases in the Notifiable Diseases Information System and are characterized as: overuse of muscles, inflammatory and/or degenerative musculoskeletal disorders, repetitive movements, and improper postures (which can lead to temporary or permanent disability), and suffering [5]. Artisanal fishers are among the most vulnerable populations due to the physical demands of their work (catching, storing, transporting, cleaning, and selling fish) [6, 7]. Furthermore, fishing is considered one of the most dangerous occupations in the world and interacts with ergonomic, physical, chemical and biological aspects [8, 9], as well as stressor variables related to the commercialization process (fishing selling) [10].

Studies that have examined artisanal fishers show that work-related musculoskeletal disorders have a negative impact on quality of life [10–12]. However, no study has described the body regions commonly affected by work-related musculoskeletal disorders. We know that it is difficult to study the vulnerable population because there are few studies in this context (making sampling difficult), the population is difficult to access (it is far from university centers), and there is a large sample loss [13].

Although researching, writing, and publishing articles on vulnerable populations is challenging, BMC Public Health recently opened space for this international debate when Maciel et al. described the physical inactivity and lipid profile of vulnerable populations in traditional communities in the Legal Amazon [13]. However, no study has investigated the body regions commonly affected by work-related musculoskeletal disorders in vulnerable populations. Our hypothesis is that vulnerable populations, such as artisanal fishermen, have affected body regions due to work-related musculoskeletal disorders. Therefore, the aim of this study was to describe the common body regions affected by work-related musculoskeletal disorders in vulnerable populations (artisanal fishermen).

## Methods

### Design and context

A cross-sectional study reported according to the STROBE guidelines [14]. The fishing colonies studied were “Z 22” (Ipueiras) and “Porto Real” (Porto Nacional, TO, Brazil). Porto Nacional and Ipueiras are Brazilian municipalities in the state of Tocantins. Porto Nacional is considered a regional hub close to the capital (57 km), Palmas, being an important access to some regions of the state and the country. Ipueiras is located 107 km from the capital Palmas.

The data collection was developed by a previously trained team between the months of June/2019 and October/2022. The first contact was made with the presidents of the fishing colonies, in order to present the objectives of the research, to clarify doubts, to invite the free participation of the fishermen registered in the colony and to obtain free and informed consent.

### Participants

This study was approved by the Research Ethics Committee of the Centro Universitário Luterano de Palmas (report number: 1.416.297) and participants provided written consent prior to study enrollment. Informed consent was obtained from all subjects and/or their legal guardian(s). All methods were performed in accordance with the relevant guidelines and regulations.

We included artisanal fishermen registered in the fishing colony, aged > 18 years. All fishermen from the colonies of Porto Nacional and Ipueiras, TO, Brazil, were invited to participate in the study. Exclusion criteria were: diagnosis of other diseases (e.g., cancer, fibromyalgia, myofascial pain syndrome, history of tumors, diagnosed neurological and cognitive problems) and the presence of other disorders (acute infections or traumas, fractures, surgeries, pain related to accidents and traumatic injuries of the musculoskeletal system).

### Assessments

Socioeconomic conditions were assessed using the Economic Classification Criteria Questionnaire proposed by the Brazilian Association of Research Companies. This criterion takes into account the possession of household conveniences, the education of the head of the family and the characteristics of the household, and classifies the economic class of the families into eight strata: A1, A2, B1, B2, C1, C2, D, E, as described by Maciel et al. [13].

The Nordic Musculoskeletal Disorder Questionnaire was developed to identify and characterize reports of musculoskeletal symptoms. The scoring followed the criteria proposed in the instrument, measuring frequencies and percentages for affected body regions. It represents one of the most important instruments used in an occupational or ergonomic health context and has shown good validity as a measure of musculoskeletal morbidity [15].

The World Health Organization Quality of Life (WHOQOL-bref) consists of 26 questions, of which 2 are general questions about quality of life and 24 are divided into 4 domains: physical, psychological, social relationships and environment. The scores range from 0 to 20 for each WHOQOL-bref domain, composed of options with values from 1 to 5 on a positive Likert scale. The scores are calculated using the syntax provided by the WHO. The closer to 0, the worse the perceived quality of life, and the closer to 20, the better the perceived quality of life in that domain [16].

Horner and Hostberg’s questionnaire is composed of 19 Likert-type questions with a total score ranging from 16 to 86 points to assess chronotype (self-report questionnaire to determine morning-evening in human circadian rhythms), i.e., the time of day the individual prefers to perform daily activities. A score of 16 to 30 points identifies an evening person; a score of 31 to 41 points identifies a moderately evening person; a score of 42 to 58 points identifies a neutral/no type; a score of 59 to 69 points identifies a moderately morning person; and a score of 70 to 86 points identifies a morning person. The final score is the result of the arithmetic sum of each point associated with the answer given in each question [17, 18].

The Perceived Stress Scale (PSS-10) is an instrument that measures the degree to which situations in an individual's life are perceived as stressful through 10 self-report questions. Six items on the scale are negative (1, 2, 3, 6, 9, 10) and the remaining four are positive. Each item is rated on a five-point Likert scale (1 = never to 5 = very often). To create the score, the four positive items are consolidated as reverse scores and then all items are summed, with scores ranging from 0 to 40. A higher score indicates greater stress. The final result, although not a criterion-referenced measure, can be compared to reference populations [19].

### Statistical analysis

To reduce the possibility of information bias, we provided prior training in the use of the instruments and created an electronic database (Excel®) that was completed in duplicate, with a third researcher consulted in cases of disagreement. We checked the normality of the data using the Shapiro–Wilk test and presented them as means, standard deviations and medians. For data analysis, frequency distribution tables were constructed and measures of central tendency were calculated according to the numerical distribution found. A significance level of 5% was considered using SPSS® software, version 17.0 (Chicago, IL, USA).

## Results

The sample consisted of 132 participants, but there was a sample loss of 41.6% (withdrawal). Therefore, the final sample is composed of 77 artisanal fishermen, most of them male (61.04%), single and working from 6 to 8 h per day. All the artisanal fishermen use hooks, fishing rods, canoes, small boats and nets in their work routine. Table 1 describes the socio-demographic characteristics of the sample. Regarding the chronotype, we observed that the median is between 42 and 53 (median = 50), a value that corresponds to the neutral pattern (i.e., neither morning nor afternoon). Table 2 describes the working conditions. We observed the predominance of those who work from 6 to 8 h/day (40.91%), rest of 1 h during the working day (45%), from 1 to 10 years of service (59.46%), and only 1 employment relationship (82.61%).

**Table 1:** Sample characteristics (*n* = 77)

Variables	n (%)
Sex	
Male	47 (61.04)
Female	30 (38.96)
Son	
0	10 (13.89)
1	6 (8.33)
2	11 (15.28)
≥ 3	45 (62.50)
Companions	
No	44 (59.46)
Yes	30 (40.54)
Economic class	
B2	4 (5.19)
C1	10 (12.99)
C2	29 (37.66)
D-E	34 (44.16)

**Table 2:** Work conditions (*n* = 77)

Variables	n (%)
Workday (h/day)	
from 6 to 8	27 (40.91)
from 10 to 12	18 (27.27)
> 12	21 (31.82)
Rest (h)	
0	15 (21.13)
1	32 (45.07)
2	24 (33.80)
Service time (years)	
< 1	14 (18.92)
from 1 to 5	23 (31.08)
from 6 to 10	21 (28.38)
from 11 to 19	10 (13.51)
> 20	6 (8.11)
Number of jobs	
1	57 (82.61)
2	11 (15.94)
3	1 (1.45)

Table 3 describes the quality of life and stress levels. We observed a worse result in the domain related to the environment. The higher the score, the better the quality of life, ranging from 0 to 20. The stress level was 15.43 (± 7.52) with a maximum of 30 points. Table 4 describes the general analysis of the data obtained through the Nordic Musculoskeletal Disorders Questionnaire. We observed the presence of pain self-reported by the artisanal fishermen in several regions, the lumbar (29.87%) being the most frequently mentioned.

**Table 3:** Quality of life and perceived stress (*n* = 77)

Variables	Mean (standard-deviation)	Minimum (maximum)
Quality of life		
Physical	15.51 (2.19)	7.43 (20.00)
Psychological	15.63 (2.12)	10.40 (19.33)
Social relationships	15.92 (2.37)	9.33 (20.00)
Environment	13.01 (2.13)	9.50 (18.50)
Self-evaluation	14.63 (2.49)	10.00 (20.00)
General quality of life	14.72 (1.81)	8.17 (18.77)
Stress level	15.43 (7.52)	1 (30)

**Table 4:** Commonly affected body regions from work-related musculoskeletal disorders in artisan fishermen (*n* = 77)

Body region	No pain, n (%)	Rarely, n (%)	Often, n (%)	Always, n (%)
Neck	43 (55.84)	15 (19.48)	12 (15.58)	7 (9.09)
Shoulders	50 (64.94)	9 (11.69)	9 (11.69)	9 (11.69)
Arms	52 (67.53)	10 (12.99)	7 (9.09)	8 (10.39)
Elbows	68 (88.31)	4 (5.19)	1 (1.30)	4 (5.19)
Forearm	66 (85.71)	6 (7.79)	2 (2.60)	3 (3.90)
Wrists	57 (74.03)	5 (6.49)	8 (10.39)	7 (9.09)
Back	38 (49.35)	11 (14.29)	12 (15.58)	16 (20.78)
Lumbar	21 (27.27)	14 (18.18)	19 (24.68)	23 (29.87)
Lower limbs	41 (53.25)	10 (12.99)	13 (16.88)	13 (16.88)

## Discussion

Prevalence of work-related musculoskeletal disorders among artisan fishermen is high, lumbar (29.87%) and dorsal (20.78%), indicating that body regions report the presence of musculoskeletal symptoms. Studies carried out in other countries report that low back pain is the most frequent symptom among artisan fishermen. A study carried out in Asia reports a percentage of 77% of work-related musculoskeletal disorders in small-scale artisan fishermen, 61% of low back pain, and 37% of shoulder pain. Repetitive movements, high loads, inadequate tools and environment, and the amount of time in the profession are decisive for the development of these disorders [19, 20].

Present study demonstrated that the lumbar/dorsal regions and the lower limbs are the most affected by work-related musculoskeletal disorders. This result is important because, when untreated, the worsening and progression of these pains and musculoskeletal injuries are observed. In addition, significant levels of stress and a perceived decline in quality of life were demonstrated. The preservation of health is essential for the worker to maintain his work activity and his livelihood, with health promotion and prevention measures being a priority [21].

Fishermen’s work is basically manual and carried out without any (or almost no) protection. As there is no basic prevention and technologies to help transport heavy loads, the occurrence of injuries among these workers is frequent. According to the result of the Fourth European Working Conditions Survey, fishing workers are among those most exposed to danger with repetitive movements and high loads during their work. Danish research reported preventive measures and modernization of materials and fishing equipment, facilitating ergonomic loads and reducing diseases, a reality far from the Brazilian reality, in which fishermen spend excessive hours at work to be able to bring their fish and sell it [19, 22].

Another research showed that the construction of an automatic fishing net achieved a risk reduction of 64.5% for work-related musculoskeletal disorders, showing the importance of applying interventions aimed at the work process. However, there is little research available on this subject, which is so relevant for both fisheries workers and institutional policies [19].

In the United Kingdom, a survey highlighted the need for health promotion services aimed at the specific conditions of this community. Cultural and social factors directly influence adherence to health services. It was reported that men are more reluctant to seek health care, creating barriers and, therefore, requiring interventions to change attitudes. Identifying the real needs of the community would imply a greater reach of this population, thus improving the health and effectiveness of policies aimed at this population [10].

Quality of life is a very subjective factor, as improving people's living conditions does not necessarily result in an increase in their levels. However, it is possible to detect some problems and make interventions that can stimulate the change of harmful behaviors in a community, making these individuals receive and achieve means to improve their living conditions [23]. Having a good perception of quality of life is considered important, but it is related to what each individual considers to be relevant and sufficient for their life. It can be seen that the importance and satisfaction of individuals can be transformed into a collective benefit. Therefore, it is understood that prevention and health promotion interventions are necessary in all its determinants [24, 25].

Artisan fishermen’s quality of life is impaired when there are work-related pathologies, with reports that artisanal fishermen/shellfish gatherers are among the classes most affected by work-related musculoskeletal disorders. This association between fishing work and the incidence of injuries directly impacts health, considering that this occupation has an informal condition [26]. Another study on artisan fishermen and factors associated with quality of life, it was found that 12 h or more of work has a negative impact on quality of life, both in the physical and psychological domains [20].

Lowest scores achieved were in the environment domain, referring to the facets: physical security and protection, home environment, financial resources, health, and social care: availability and quality, opportunities to acquire new information and skills, recreation and leisure, physical environments such as pollution, noise, traffic and climate and transport, suggest that these factors stand out, causing discomfort in the perception of workers. These findings are in line with what is described in the literature, which highlights the lack of structured and permanent actions for worker health surveillance aimed at the category [26].

Lack of control over all aspects of work also results in high levels of stress [22]. This study showed considerable levels of stress among artisan fishermen, higher than in the American population pointing to the presence of pain and musculoskeletal disorders as one of the factors possibly involved in its occurrence and/or aggravation [27].

Studies link the chronotype with the worker’s quality of life. Several factors such as sleep disorders, tiredness, and impairment of cognitive functions may be related to work shifts. When workers do not reconcile their preferred schedules with those of work, they are subject to instability in their biological rhythm and production, interfering with their quality of life [20, 28, 29]. This work was compatible with the literature, having demonstrated that artisan fishermen were neutral/intermediate in terms of chronotype, as they have greater flexibility in the allocation of sleep–wake times, and can perform their tasks at any time of the day.

Working and health conditions of artisanal fishermen are often neglected. There is omission and fragility in relation to this class of workers, in addition to the scarcity of effective institutional policies [30]. Artisan fishermen need comprehensive care for their health. It is necessary to know the work environment, guide them, and propose concrete changes and rehabilitation. These health problems are also related to the quality of life of these workers [20, 31].

In view of these considerations, the health risks in artisanal fisheries and the absence of public policies are facts reported and verified in scientific research, but it is worth noting that the official statistical data of Brazilian fisheries are outdated, being neglected, and requiring a closer look at this population [13]. Results of this work indicate the need to approach the work process of artisan fishermen, whose lack of activity and adequate means can bring risks and damage to health. They also signal the urgency of specific public policies for the sector, aiming at improving the quality of work and the quality of life of fishermen and their families.

This study has important limitations. The sample consisted of artisanal fishers from two Brazilian cities. Therefore, there are limitations in terms of population representation (considering the total number of artisanal fishermen registered in the country). Also, there is no research with the same objective as the study, which limits the comparability of the results. We suggest that future studies should further analyze possible differences between male and female fishers and whether disorders vary according to age.

## Conclusion

The neck, shoulders, arms, elbows, forearms, wrists, back, lumbar spine, and lower limbs are the most common parts of the body affected by work-related musculoskeletal disorders in artisanal fishermen.

## Data Availability

The data and materials in this paper are available from the corresponding author on request.
